# Diet and the epigenome

**DOI:** 10.1038/s41467-018-05778-1

**Published:** 2018-08-28

**Authors:** Yi Zhang, Tatiana G. Kutateladze

**Affiliations:** Department of Pharmacology, University of Colorado School of Medicine, Aurora, CO 80045 USA

## Abstract

Over the past decade, remarkable breakthroughs in our understanding of epigenetic biology have coincided with an increased public interest in the impact of diet and lifestyle choices on health. It is well established that a balanced diet enhances life expectancy and helps to prevent or treat certain diseases, such as obesity, diabetes, cancer, and mental disorders. However, the biological mechanisms underlying these effects are not yet well understood. In this commentary, we highlight several recent studies that report on a potential link between dietary factors and alterations in epigenetic pathways, providing compelling insight into the possible effects of environmental factors on fundamental biological processes.

Two major elements of the human epigenome are covalent chemical modifications present on DNA and histones that define chromatin structure and are referred to as epigenetic marks. Epigenetic marks do not change DNA sequence, and therefore the entire genomic information or genotype, inherited from our parents remains untouched. However, epigenetic marks transform the local chromatin environment and thus affect DNA accessibility and regulate a wide range of DNA-templated processes, including gene transcription. Once misplaced or aberrantly active, epigenetic marks can disrupt normal gene expression profiles, incorrectly turning genes on and off. Dozens of epigenetic marks on histones and several on DNA have been identified, with methylation and acetylation of histones and methylation of DNA being the most prevalent^1^. Unlike the fixed DNA sequence, epigenetic marks are less stable and can undergo changes during the cell cycle or in response to various stimuli required for normal cell growth and survival.

A wealth of recent studies suggests that epigenetic marks are also sensitive to environmental exposure. Nutrients, toxins, pollutants, pesticides and other environmental factors can impact, either directly or indirectly, levels and turnover of epigenetic marks. This in turn would result in transformed gene expression patterns and consequently affect our health for better or worse. Certain epigenetic alterations have been thought to pass to the next generation or cause a temporal modulation of subsets of genes eventually leading to progression of disease^2–5^. Three studies recently published in *Nature Communications* have explored how diet or compounds found in food can alter gene expression programs through epigenetic mechanisms, opening a new avenue in exploring therapies based on these mechanisms.

How could food consumption influence epigenetic modifications that would eventually make an impact on individual health? One possibility is through directly affecting catalytic activities of the enzymes responsible for ‘writing’ or ‘erasing’ the epigenetic modifications. Wang et al. identified two phytochemicals—dihydrocaffeic acid (DHCA) and malvidin-3′-O-glucoside (Mal-gluc), metabolic intermediates derived from Concord grape juice, grape seed extract, and trans-resveratrol—that attenuate depression-like behaviors in mice^6^. Using a mouse model, Wang et al. showed that treating mice with DHCA and Mal-gluc, which were added to drinking water, increased their resilience to stress and reduced depression-like behaviors. Specifically, the authors found that DHCA reduced expression of “methyl-DNA writer”—the DNA methyltransferase 1 (DNMT1). DNMT1 methylates intronic sequences of interleukin 6 (*IL-6*) genes, reducing level of this pro-inflammatory cytokine which has previously been implicated in the development of depressive disorders. Mal-gluc on the other hand reduced the expression of an “acetyllysine eraser”, histone deacetylase 2 (HDAC2), which led to a significant increase in histone H3 acetylation on promotor of the RAS-related botulinum toxin substrate 1 (*Rac1)* gene, whose expression level was found to be downregulated under chronic stress. If combined, the treatment of DHCA and Mal-gluc reduced IL-6 level to baseline and simultaneously increased *Rac1* expression, which contribute to the resilience against the development of depression-like phenotypes in mice. Overall, the study by Wang et al. provides striking evidence of how grape-derived phytochemicals can influence inflammation and brain synaptic plasticity, promoting resilience against stress in mice. This raises the possibility that these compounds, perhaps in combination with currently available antidepressants, could be of interest as therapeutic candidates in the treatment of patients suffering from depression and anxiety.

Another epigenetics-driven mechanism involves metabolically generated intermediates that are capable of hijacking histone residues normally modified by more conventional epigenetic marks. Huang et al. reported on a new epigenetic modification, histone lysine benzoylation, identified by HPLC-MS/MS analysis first in human cells and then in mouse liver and *Drosophila* cells^7^. Metabolic labeling assays performed in cells in vitro showed that benzoyllysine is generated from a co-factor benzoyl CoA, which is likely a product of chemical reaction involving the organic compound sodium benzoate. Sodium benzoate is an FDA approved preservative, commonly used as an antimicrobial and flavoring agent to prevent canned fruit and fruit beverages from molding. ChIP-seq and RNA-seq analyses presented by Huang et al. revealed that the lysine benzoylation mark is enriched on promoters of active genes, particularly those implicated in glycerophospholipid metabolism, phospholipase D signaling, ovarian steroidogenesis, serotonergic synapse, and insulin secretion. Furthermore, Huang et al. found that in contrast to other known acyllysine modifications that are “erased” by several HDACs, the lysine benzoylation mark can be removed only by SIRT2, a NAD^+^-dependent protein deacetylase. The latter finding suggests that benzoyllysine level is dynamic and can be fine-tuned in a manner independent of histone acetylation. Although the question of whether dietary sodium benzoate can lead to epigenetic changes in vivo remains open, the study by Huang et al. indicates a possible effect of the compound on lysine benzoylation in cultured cells. Sodium benzoate-induced histone lysine benzoylation therefore represents a potential example of an epigenetic mechanism in which metabolic products could directly influence epigenetic modifications that affect gene expression.

Malnutrition in early life has directly and indirectly been linked to a number of disorders in adulthood, giving rise to the hypothesis of epigenetic memory. In their study, Yuan et al. showed that lactation in mice causes epigenetic changes that influence the likelihood of the development of obesity later in life^8^. Milk lipids are known to activate the nuclear receptor, PPARα (peroxisome proliferator-activated receptor), an important transcriptional regulator of hepatic liver metabolism. Using a genome-wide analysis of DNA methylation, Yuan et al. identified several PPARα target genes, including the fibroblast growth factor-21 (*Fgf21*), that undergo PPARα-dependent DNA demethylation upon pharmacological activation of PPARα in mice. Fgf21 is a liver hormone essential in a number of metabolic functions, including body weight maintenance and regulation of energy homeostasis. Yuan et al. found that once established in early life, the DNA (de)methylation status of Fgf21 remains unchanged in the adulthood, and that the reduced DNA methylation of *Fgf21* correlates with a reduction in diet-induced obesity in older animals. The authors further demonstrated that *Fgf21* demethylation is stimulated during the lactation period of newborn mice. The results of this study are striking and may have a profound impact on our understanding of the mechanisms contributing to obesity. These data suggest a link between breastfeeding and weight gain suppression and also elegantly illustrate how specific epigenetic modifications built in early life produce a long-lasting effect.

Many other factors have been shown to markedly contribute to changes in epigenetic programs and responses. For example, Krautkramer et al. reported that gut microbes and their metabolites affect host chromatin state, increasing histone poly-acetylation and producing short-chain fatty acids (SCFAs)^9^. A “Western-type” diet rich in processed foods and high sugar drinks was found to limit microbial SCFAs production, prevent many of the microbiota-dependent events to occur, and lead to alterations in hepatic gene expression. Another example is the effect of a low-carb ketogenic diet that was shown to rescue hippocampal memory defects in a mouse model of Kabuki syndrome, characterized by loss of site-specific histone methylation and deficiency in chromatin opening^10^. This diet promotes formation of β-hydroxybutyrate, an HDAC inhibitor, and leads to changes in H3ac and H3K4me3 in the hippocampus and rescue of the neurogenesis and memory phenotypes of these mice models.

It is inspiring to witness a gigantic leap in gaining knowledge of biological, mechanistic, and physiological aspects of epigenetics. Although much has been learned, we have only really scratched the surface in understanding the relationship between the fascinatingly complex human epigenome and environmental factors. The studies discussed above highlight potential roles of the environmental epigenetic changes that have to be put into the equation as we attempt to draw a comprehensive map of epigenetic networking. It will be exciting to see more studies dissecting the effect of dietary components on epigenetic imprinting, exploring alternative nutrition-based therapeutic approaches, and developing tools for personalized diet to improve health and increase life expectancy.
